# Synthesis and characterization of polyethylene glycol-phenol-formaldehyde based polyurethane composite

**DOI:** 10.1038/s41598-019-56147-x

**Published:** 2019-12-20

**Authors:** Juan Liu, RiQing Chen, ChunPeng Wang, YongJun Zhao, FuXiang Chu

**Affiliations:** 1 College of Biological, Chemical Science and Engineering, Jiaxing University, Jiaxing, 314001 China; 2 Institute of Chemical Industry of Forestry Products, CAF, Nanjing, 210042 China; 3 Chinese Academy of Forestry, Beijing, 100091 China

**Keywords:** Polymer characterization, Polymers

## Abstract

A series of phenol-formaldehyde-polyethylene glycol polyether polyols (PF-PEGs) were synthesized through the condensation polymerization and etherification of phenol, formaldehyde, and poly(ethylene glycol) (PEG) under alkaline conditions and subsequently reacted with 1,6-hexamethylene diisocyanate to obtain polyurethane (PU) films using acetone as solvents. The influence of phenol and formaldehyde to PEG mass ratio ((P + F)/PEG) on the hydroxyl number of PF-PEGs and mechanical properties, thermal stabilities, crystallization behaviors, as well as microstructure of polyurethane composite films were studied using chemical analysis, mechanical tests, thermogravimetric analyses (TGA), dynamic mechanical analyses (DMA), X-ray diffraction (XRD), scanning and transmission electron microscopies (SEM and TEM), respectively. Results demonstrated that PF-PEGs with (P + F)/PEG of 50/50 had the highest hydroxyl number of 323 mg K(OH)/g. The incorporation of phenol and formaldehyde into PEG improved the mechanical properties of polyurethane films, glass transition temperature (*T*_g_), and thermal properties but resulted in the brittleness characteristic of the composite films and low crystallization properties. Moreover, the synthesis mechanism of PF-PEGs polyurethane composite films was revealed, which would provide a theoretical base for the preparation of the rigid polyurethane foams based on phenolic resins.

## Introduction

Phenolic foams are prepared with phenolic resins that are synthesized by the condensation polymerization between phenol and formaldehyde under alkaline conditions. The foams have many advantages, such as excellent thermal insulation properties, flame resistance, and low smoke emission^1^. In recent years, enhancement of the toughness of phenol foams has received tremendous attention because of its low strength, extreme brittleness and friability. Phenolic resins (PF) are toughened mainly using flexible polymers, such as polyurethane prepolymer^2^, cardanol^3,4^, polyether polyols^5,6^, epoxy^7^, rubber^8–10^. Besides, renewable biomass, such as tannin^11^, natural fibres^12,13^, have been also used to modify PF resin matrix. Although the mechanical properties of foams have been improved by toughening technique, the equipment is easily corroded during the production and use process of foams because of acid curing agents. Rigid polyurethane (PU) foams that prepared with polyether polyols and isocyanate have desirable mechanical property but poor flame retardant performance^14^. Using isocyanate as curing agents and phenolic resin matrix as polyether polyols to prepare PU composite materials has an important significance for obtaining foams with excellent mechanical and flame retardant properties.

Polyurethane materials are mainly prepared through addition reaction between hydroxyl groups of polyols and isocyanate groups. The types of polyols, segment length distribution, crystallization, and intersegment interactions affect the mechanical properties, thermal properties, and domain morphologies of thin films contain^15^. The polyols used to prepare PU materials usually include poly(ethylene glycol) (PEG)^16^, poly(ethylene oxide) (PEO)^17^, poly (tetramethylene oxide)^15^, and poly(propylene glycol) (PPG)^18^. It is well known that phenolic resins contain reactive hydroxyl methyl and hydroxyl groups that react with isocyanate groups to form three-dimensional polyurethane networks, providing a theoretical base for the preparation of the polyurethane foams based on resole phenolic resins. The effects of polyethylene glycol (PEG) on mechanical property, microstructure, thermal stability, and flame resistance of phenol–urea–formaldehyde foams were investigated using PEG only as toughening agent, and acid rather than isocyanate as curing agents^19^. Many researches on the combination of polyurethane and phenolic resins have been reported, but most of the polyurethane materials used are PU prepolymers that are blended with phenolic resin to improve toughness of the foams^20^. There are rare studies that focused on the incorporation of phenol and formaldehyde into PEG to prepare polyurethane composite.

In this work, phenol, formaldehyde, and poly(ethylene glycol) were used to synthesize phenol-formaldehyde-polyethylene glycol polyether polyols (PF-PEGs) by condensation polymerization and etherification reaction. And the PU films were prepared with the synthetic PF-PEGs and 1,6-hexamethylene diisocyanate (HDI), using acetone as solvents to reduce viscosity and form films through solvent volatilization. The objective of this work was to synthesize PF-PEGs, prepare PU films and investigate the effects of phenol and formaldehyde to PEG mass ratio ((P + F)/PEG) on mechanical properties, thermal stability and microstructure of films. Fourier transform infrared spectroscopy (FT-IR) was used to characterize PF-PEGs. And the mechanical properties, thermal stabilities properties, crystallization behaviors, and micromorphologies of PU films were studied by mechanical tests, dynamic mechanical analysis (DMA), thermogravimetric analysis (TGA), X-ray diffraction (XRD), scanning and transmission electron microscopies (SEM and TEM), respectively. Moreover, the formation mechanism of PF-PEGs polyurethane composite films was explored.

## Materials and Methods

### Materials

Poly(ethylene glycol) (PEG) of 400 g·mol^−1^ was supplied by Shanghailingfeng. Chemical Reagent CO., Ltd and distilled at 110 °C for 4 h under vacuum condition. Phenol, paraformaldehyde, formic acid, sodium hydroxide and acetone were provided by Nanjing. Chemical Reagent Co., Ltd. Acetone was dried with 4 A molecular sieves before utilization. 1,6-hexamethylene diisocyanate (HDI, 99%, aladdin) was obtained from commercial resource and used as received.

### Synthesis of PF-PEGs

**Figure 1:**
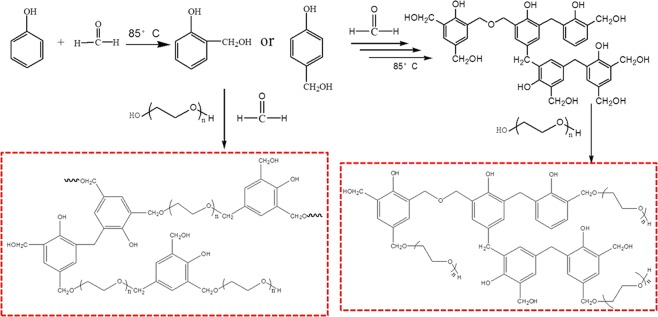
The synthetic route of PF-PEGs.

A series of PF-PEGs were synthesized through condensation polymerization with different (P + F)/PEG mass ratio, which were showed in Table 1. The molar ratio of formaldehyde to phenol (F/P) was 1.8. PEG and melted phenol were loaded into a 250 mL round-bottomed flask equipped with a stirrer, a thermometer, a reflux condenser. The pH of system was adjusted to around 11.5 with NaOH solution (50 wt.%). Keeping the temperature at 65 °C, paraformaldehyde was divided into four parts and added into the flask every 15 min. When all of the paraformaldehyde was added into the flask, the system was slowly heated to 85 °C for 4.5 h. During the reaction process, pH was constantly adjusted with NaOH solution to maintain around 11.5. The reaction mixture was cooled to 45 °C and the pH was adjusted to 7.0 with formic acid. Finally, the water in the mixture was removed by reduced pressure distillation. PF-PEGs with different (P + F)/PEG mass ratio were synthesized as shown in Fig. 1.

**Table 1:** Compositions of PF-PEGs and PU films prepared with polyether polyols and HDI.

PU films	Polyether polyols	(P + F)/PEG mass ratio	F/P molar ratio	Isocyanate index
PM-1	PEG	0/100	0	1.05
PM-2	PF-PEGs-1	10/90	1.8	1.05
PM-3	PF-PEGs-2	20/80	1.8	1.05
PM-4	PF-PEGs-3	33/67	1.8	1.05
PM-5	PF-PEGs-4	40/60	1.8	1.05
PM-6	PF-PEGs-5	50/50	1.8	1.05

### Preparation of PU films

The prepared PF-PEGs dissolved in acetone was charged into a 100 mL four-necked round-bottomed flask equipped with a magnetic stirrer, a nitrogen inlet and thermometer. The mixture was stirred at 70 °C under nitrogen environment until the complete dissolution. The HDI previously dissolved in acetone was added dropwise into the dissolved PF-PEGs. The mixture was stirring for 2 h at 70 °C. The content of PF-PEGs and HDI were calculated on the basis of the isocyanate index (the NCO: OH molar ratio). After completion of the reaction, the mixtures were poured into a Teflon mold and then placed at room temperature for 12 h. Subsequently, the obtained films were transferred to vacuum drying oven at 60 °C for 5 h to remove solvent completely. The prepared PU films were named from PM-1 to PM-6, which were displayed in Table 1.

### Characterization of PF-PEGs and PU films

Fourier transform infrared (FT-IR) spectrometry of PF-PEGs and PEG (after freeze drying) was recorded on a Nicolet IS10 FT-IR spectrometer. The hydroxyl number of PEG and PF-PEGs was measured in accord with the ASTM standard 6342-08. Tensile strength of PU films was obtained from a SANS CMT 4000 universal testing machine. The dynamic mechanical analysis of films was carried out on a DMA Q800 (TA instruments) with tension model at a fixed frequency of 1 Hz under nitrogen and the films samples (40 mm × 6 mm × 0.6 mm) were heated from −140 to 150 °C with a heating rate of 3 K/min at ambient temperature (25 ± 1) °C. The X−ray diffraction (XRD) measurements of the films were performed in a Shimadzu 6000 diffractometer with radiation CuKa (k = 15.4 nm, 40 kV and 40 mA) at 25 °C. The relative intensity was registered in a dispersion range (2*θ*) from 10 to 80 ° at a step size of 0.04 °. TGA measurements were performed using NETZSCH (Germany) STA 409 apparatus with a nitrogen flow of 20 mL/min. Approximately 5 mg of each polyurethane film was measured from 35 to 600 °C with a heating rate of 10 °C/min. The microstructure morphologies of films were observed by scanning electron microscopy (SEM, S-3400N, Hitachi co., Japan). The samples were broken under liquid nitrogen environment and the fracture surface was used for observation. The transmission electron microscope (TEM) observation of morphology of composite polymer films was performed using a TECNAI G2 20 instrument at an accelerating voltage of 200 kV. The films were prepared by ultramicrotomy of frozen section and the thickness of sections was 70 nm.

## Results and Discussion

**Figure 2:**
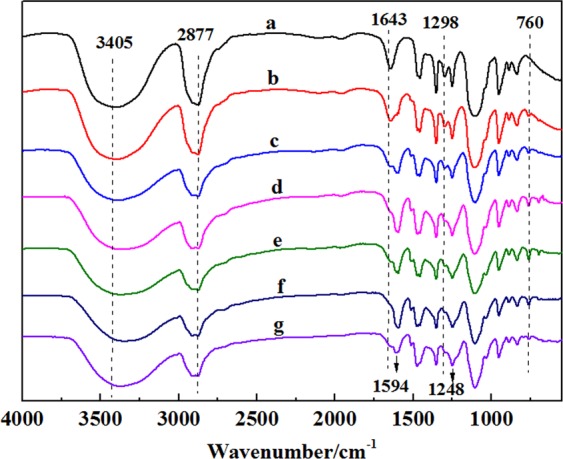
FT-IR analysis of freeze-dried PEG, PF-PEGs, and PEG/PF blends in the range of 4000–550 cm^−1^. (**a**) FT-IR spectra of PEG. (**b**–**f**) FT-IR spectra of PF-PEGs-1, PF-PEGs-2, PF-PEGs-3, PF-PEGs-4, PF-PEGs-5, respectively. (**g**) FT-IR spectra of PEG and PF blends. Plot at the top and low shows the wavenumber of bands of PEG, PF-PEGs, and PEG/PF blends.

The *ortho-* and *para-* hydrogens with high activity took place condensation reaction with formaldehyde forming hydroxymethyl groups. And plenty of hydroxyl groups in PEG reacted with hydroxymethyl groups of low-condensation resols through etherification. Figure 2 shows the representative bands assignment of PEG, PF-PEGs, and PEG/PF blends (mass ratio of 1.0:1.0). FT-IR spectrum showed a broad absorption band in the region of 3200−3550 cm^−1^, corresponding to stretching vibration of hydroxyl groups. Compared with PEG, the broad peak of PF-PEGs shifted to lower wavenumber with the formaldehyde and phenol contents increasing due to the phenolic hydroxyl and newly formed hydroxymethyl groups between formaldehyde and phenol. The peak at 2877 cm^−1^ was assigned to stretching vibration of –CH_2_– group, while 1643 cm^−1^ linked to the hydroxyl groups^21^. The hydroxyl band at 1643 cm^−1^ became weaker with the increase of phenol and formaldehyde contents, which was because that the hydroxyl groups of PF-PEGs decreased by etherification reaction of hydroxyl groups and hydroxymethyl. And PEG/PF blends had a weak absorption band at 1643 cm^−1^. The difference of absorption band between PF-PEGs-5 and PEG/PF blends indicated that the hydroxyl groups of PEG indeed took part in etherification reaction with hydroxymethyl groups. The new peak at 1594 cm^−1^ of PF-PEGs was attributed to aromatic ring vibrations and the intensity of the peak increased significantly with the decrease of PEG contents. The bands at 1298 cm^−1^ and 1248 cm^−1^ were related to asymmetric stretching vibration of C–O and C–O–C, respectively. Compared with the FT-IR spectra of PEG, the peak at 1298 cm^−1^ of PF-PEGs disappeared and the peak at 1248 cm^−1^ became wider that were attributed to etherification. The PF-PEGs showed a new peak at 760 cm^−1^, which indicated the substitution of *ortho*-hydrogen of phenol.

**Figure 3:**
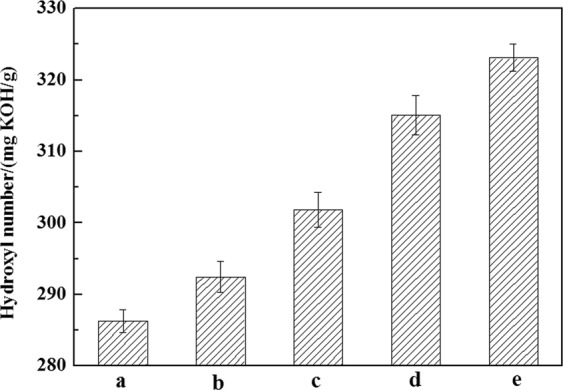
Effects of (P + F)/PEG mass ratio on the hydroxyl number of PF-PEGs. (**a**) Polyether polyols of PF-PEGs-1. (**b**) Polyether polyols of PF-PEGs-2. (**c**) Polyether polyols of PF-PEGs-3. (**d**) Polyether polyols of PF-PEGs-4. (**e**) Polyether polyols of PF-PEGs-5. Each treatment was done in triplicates. Graph shows average number of three sets of parallel samples.

Figure 3 shows the hydroxyl number of PF-PEGs. Phenol and formaldehyde contents had significant effects on the hydroxyl number of PF-PEGs, which could be seen from the incremental increase in hydroxyl number exhibited in Fig. 3. The dosage of curing agent HDI was calculated on the basis of hydroxyl number. The hydroxyl number of PF-PEGs increased with the incorporation of phenol and formaldehyde into PEG, which was because that more hydroxymethyl groups were formed by the condensation reaction of formaldehyde and phenol. The total hydroxyl number of PF-PEGs varied from 286 to 323 mg K(OH)/g with phenol and formaldehyde contents increasing and the PF-PEGs could be suitable for preparing PU composite. And more reactive sites (hydroxyl groups and hydroxymethyl groups) would result in higher cross-link density of the PU films.

**Figure 4:**
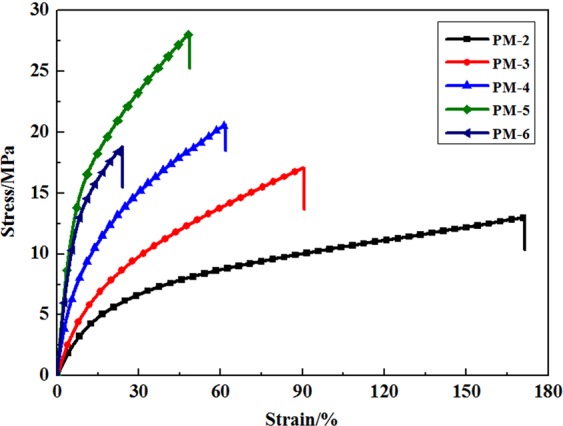
Stress-strain curves of PF-PEGs polyurethane films prepared with PF-PEGs and HDI. Please refer to Table 1 for the description of the variable acronyms.

Figure 4 shows the stress-strain curves of PU films prepared with PF-PEGs. The elongation at break for PM-1 was 728.2% with tensile stress at break about 7.4 MPa. It could be seen that tensile strength and elongation at break of PU films were dramatically affected by the incorporation of phenol and formaldehyde. PU films had higher tensile strength and lower elongation at break in comparison with PM-1. The elongation at break for PM-1 and tensile strength were 7.4 MPa and 728.2%, while that of PM-5 were 28.1 MPa and 48.7%, increasing by 279.7% and decreasing by 1395.3%, respectively. The trend resulted from rigid structure and strong hydrogen bonding effect caused by benzene ring. However, both tensile strength and elongation at break were decreased when phenol and formaldehyde contents over 40 wt% indicating that too much rigid structure of benzene ring resulted in worse mechanical properties^18^. On the other hand, compared with linear PM-1 prepared by the reaction between PEG and HDI, plenty of hydroxymethyl groups, acting as crosslink point, formed by the condensation polymerization between formaldehyde and phenol, which resulted in more complex three-dimensional polyurethane network and less flexible PEG-based chains.

**Figure 5:**
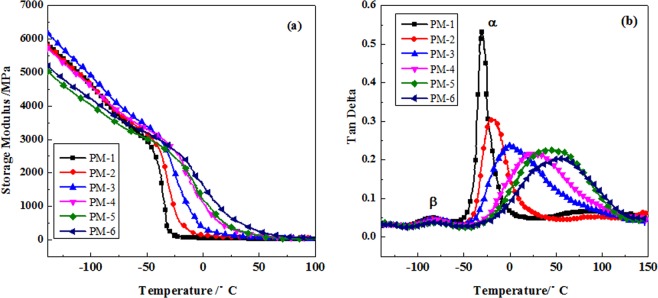
DMA curves of PU films. (**a**) The storage modulus *E*′ versus temperature of prepared PU films and (**b**) the loss factor tan *δ* versus temperature. Please refer to Table 1 for the description of the variable acronyms.

DMA experiment is performed to obtain the deformation behaviour, mechanical properties, and morphology of composites with temperature^22–24^. The storage modulus (*E*′) versus temperature of PU films observed by DMA was shown in Fig. 5(a). The degree of cross-link density could be characterized by the difference between the storage modulus (Δ*E*′) of PU films in the plateau regions before and after the glass transition. The smaller Δ*E*′ is, the greater degree of cross-link density is^25^. It could be seen from Fig. 5(a) that PU films prepared with PF-PEGs had smaller Δ*E*′ compared to that of film prepared with PEG, indicating that PF-PEGs polyurethane composites had higher degree of cross-link density. In other words, PF-PEGs polyurethane composites had higher mechanical strength than PM-1, which was consistent with the results of stress-strain test. Figure 5(a) also showed that the incorporation of phenol and formaldehyde to PEG made *E*′ decreased much more slowly in the glass transition region. The *E*′ increased with increasing concentration of phenol and formaldehyde in the glass transition region. When the content of phenol and formaldehyde was 50%, the PU film (PM-6) had the highest storage modulus.

The loss factor (tan *δ*) versus temperature observed by DMA was shown in Fig. 5(b). Tan *δ* could be used to characterize the homogeneity of composite polymers, and temperature associated with tan *δ* reflected the glass transition temperature (*T*_*g*_)^25,26^. It could be seen in Fig. 5(b) that films had two main loss peaks *α* and *β* in the temperature range of −100 to −50 °C and −50 to 140 °C, respectively. In the range of −100 to −50 °C, loss peaks of all samples had little difference. However, in the range of −50 to 140 °C, the PM-1 exhibited a much larger and sharper loss peak with a value of *T*_*g*_ of approximately −25 °C. The PF-PEGs polyurethane composites displayed a significantly broader transition and higher *T*_*g*_ upon increasing the content of phenol and formaldehyde due to the heterogeneity of the polyurethane composites films prepared with PF-PEGs and the confinement effect of three dimensional network structure on mobility of polyurethane segmental chains^27^. Besides, it could also be seen that the *α* relaxation shifted to higher temperature region with the increase of phenol and formaldehyde contents, indicating that rigid structure and multi-functional groups of the synthetic polymers resulted in higher *T*_g_ of films. It has been reported that broad loss transitions suggest superior mechanical properties^25^. Therefore, the results of loss factor tan *δ* also revealed that mechanical strength of PF-PEGs polyurethane composites was improved with the content of phenol and formaldehyde increasing.

**Figure 6:**
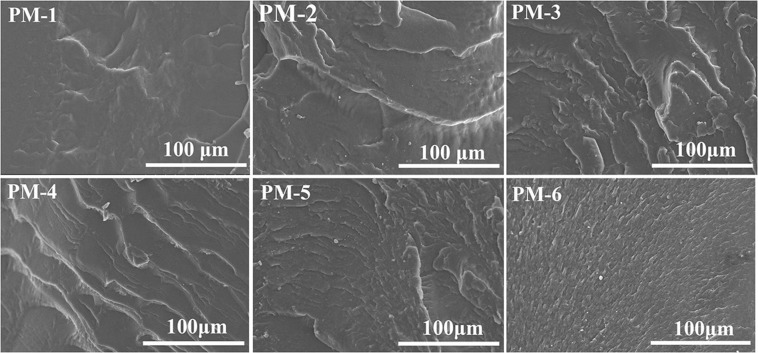
SEM images of the fracture surface morphologies for prepared polyurethane composite films. Please refer to Table 1 for the description of the variable acronyms. Scale bars for all, 100 µm.

The fracture surface morphologies of PU films were observed by SEM, as shown in Fig. 6. It was obviously that phenol and formaldehyde had effects on fracture surface of films. PM-1 presented a relatively rough fracture surface morphology, indicating a ductile fracture pattern. PM-6 exhibited a smooth and flat morphology, suggesting a brittle fracture mode. The fracture surface of PU films became much smoother and the crazing was gradually reduced as phenol and formaldehyde contents increasing, because the introduction of phenol and formaldehyde resulted in the rigidity and tensile strength were enhanced.

**Figure 7:**
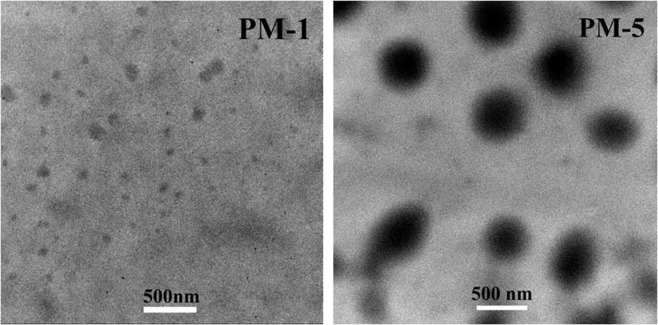
TEM images of PM-1 and PM-5 ultra-thin sections. Please refer to Table 1 for the description of the variable acronyms. Scale bars for all, 500 nm.

Macroscopic properties of PF-PEGs polyurethane composite films largely depend on their microscopic structures. TEM was conducted to investigate the effects of phenol and formaldehyde on inner morphology of PF-PEGs composite films and study the curing mechanism of PF-PEGs and HDI, and the TEM images of samples were demonstrated in Fig. 7. It has been reported that phase segregation might improve the tensile strength of polyurethane films^28^. There were some obvious microphase separation in PM-1, and the size of aggregates was small, which was because that PEG with ordered structure and flexible chains was prone to form crystallization and phase separation. The dispersed spheres with sizes of approximately 250 nm were found in PM-5 due to the *π−π* stacking interactions of rigid benzene ring structure in phenol condensation polymers or PF-PEGs copolymers. But, the rigidity of the PM-5 main-chains increased with the phenol contents increasing, which limited the mobility of segments, and was not conducive to the formation of phase separation, leading to lower crystallinity.

**Figure 8:**
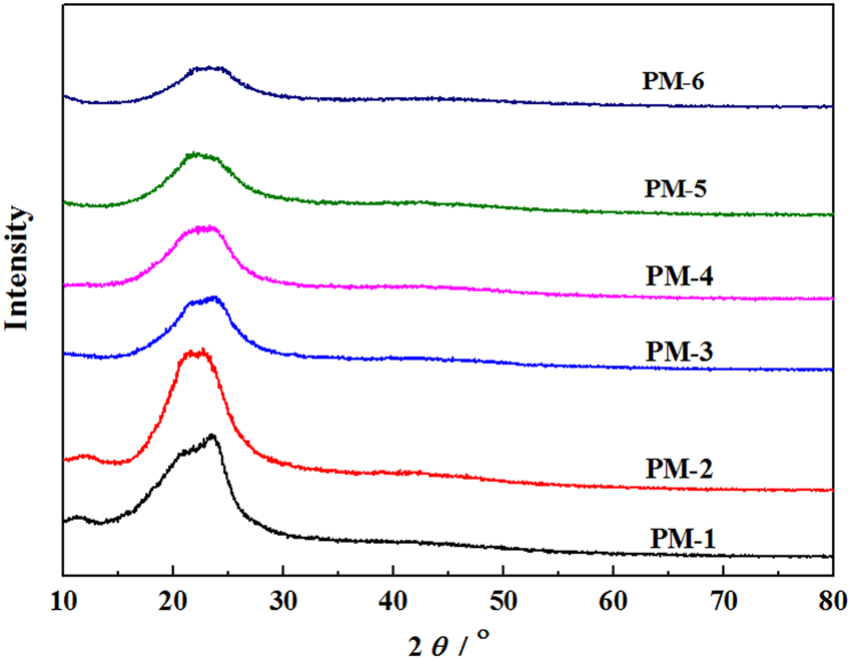
XRD patterns for PM-1 and PF-PEGs polyurethane composite films. Please refer to Table 1 for the description of the variable acronyms.

Figure 8 exhibits the X-ray diffraction patterns of polyurethane composite films. One main crystalline peak around 2*θ* = 22.5° was observed in all films, which was the paracrystalline peak of the soft segment in segmented polyurethane materials^29^. As shown in Fig. 8, all the films had a certain degree of crystallization. PM-1 had a large crystallization peak with a slight spike that was caused by the flexible and well-aligned structure of PEG molecular chains. The result was consistent with that of Behnam *et al*.^30^, who found that polyurethane product had a broad peak near 2θ = 21.7° originated from the amorphous feature of cross-link polyurethane. XRD patterns of PF-PEGs polyurethane composite films showed a weak and broad peak at 2*θ* = 22.5° and the peak was getting weaker and broader with the increase of phenol and formaldehyde contents, which indicated lower crystallinity of PF-PEGs polyurethane composite films with higher content of phenol and formaldehyde. That was because the introduction of benzene ring in phenol resulted in the complex and rigid structures, which were unfavorable for the crystallization. Besides, with the increase of phenol and formaldehyde contents, more rigid segments were dispersed in soft matrix PEG, resulting in less flexibility of molecular chains and smaller microphase separation because of the stronger hydrogen bonding between soft and hard segments.

**Figure 9:**
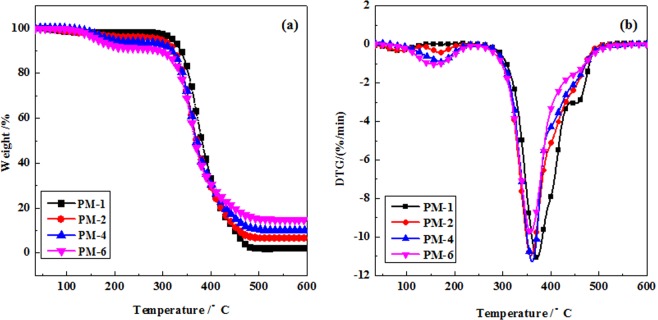
TGA (**a**) and DTG (**b**) curves for PU composite films. The thermostability was measured with a heating rate of 10 °C/min.

The thermal stability of polyurethane composites was affected by backbone structures^26,31^. Figure 9 displays the TGA and DTG curves of PU films, respectively. PM-1 showed little mass loss in 35–300 °C. And there were two degradation stages in 300–500 °C that attributed to the earlier decomposition of the hard segment and the later decomposition of the soft segments, respectively^32^. The first stage, as the main degradation stage, was caused by the decomposition of the hard segment (isocyanurate) between 300 °C and 440 °C, leading to the formation of isocyanate and alcohol, primary or secondary amine and olefin and carbon dioxide^26^. The second stage corresponded to the decomposition of soft segments (polyol segments) between 440 °C and 500 °C. For PF-PEGs composite films, a quite similar two-step thermal decomposition process was found in Fig. 9 (a,b). The phenol and formaldehyde contents affected the degradation process and carbon yield of films. The first thermal degradation process of PF-PEGs composite films below 200 °C was attributed to free phenol, free formaldehyde, and moisture evaporation produced by condensation reaction. Obviously, films had faster decomposition rate as the increased addition of phenol and formaldehyde. The second degradation step occurred at slight low temperature compared with that of PM-1, which might be because more isocyanurate (hard segment) were used in accordance with the increase of hydroxyl number of PF-PEGs copolymers that leaded to faster decomposition of the hard segment of films. The decomposition of the soft segments of PF-PEGs composite films prepared with PF-PEGs was not obvious. And the PF-PEGs composite films had slower decomposition rate in comparison with PM-1 due to the introduction of rigid benzene ring structure. Figure 9(a,b) also displays the temperature (*T*_max_) of samples with maximum decomposition rate and char residue at 600 °C. PM-1 showed *T*_max_ of 372.1 °C and the lowest char residue of only 2.1%. After hybrid network formation between PF-PEGs and HDI, PM-2 showed char residue of 6.6% at 600 °C and *T*_max_ of 359.2 °C while PM-4 had char residue of 10.0% at 600 °C and *T*_max_ of 361.9 °C. PM-6 showed *T*_max_ of 358.7 °C and the highest char residue of about 15%. The char residue of polyurethane composite films was improved with the increase of phenol and formaldehyde contents due to the high thermal stability of aromatic groups. Although *T*_max_ of PM-6 was lower than that of PM-1, PM-6 had the minimum thermal decomposition rate than other samples. Therefore, the introduction of a certain amount of aromatic structure to PEG could improve the char residue and thermal resistance of PF-PEGs polyurethane composite films.

**Figure 10:**
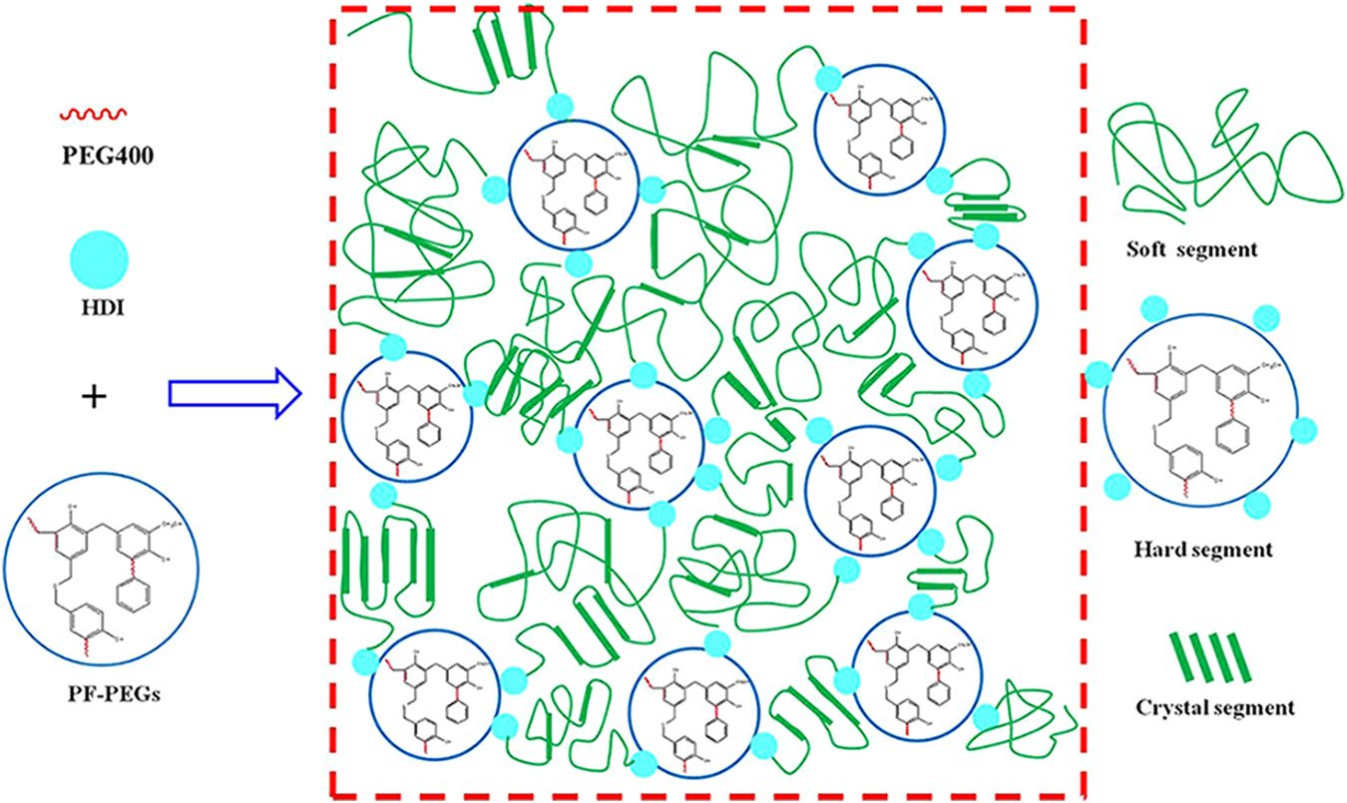
The synthesis mechanism of PF-PEGs polyurethane composite films prepared with PF-PEGs and HDI. The microscopic structures of composite films were exhibited according to analysis results discussed above.

The reaction mechanism of PF-PEGs polyether polyols and HDI for producing polyurethane composite films is exhibited in Fig. 10. It is well known that polyurethane material synthesized with PEG and isocyanate is linear structure because of two functional groups. However, on the basis of the results discussed above, PF-PEGs films had intricate structure of linear and three-dimensional polyurethane network structure including partially-crystallizaton structure and microphase separation segments. That was due to PF-PEGs had high degree of functionality formed through condensation reaction and etherification reaction. The polyols with phenyl structure acted as the cross-linking points of networks and played a predominant role for the super tensile strength.

## Conclusion

The PF-PEGs polyether polyols were synthesized successfully, which were characterized by fourier transform infrared (FT-IR) spectrometry. And hydroxyl number of PF-PEGs was improved with the increase of phenol and formaldehyde contents. The effects of (P + F)/PEG on mechanical properties, microstructure, and thermal stability of polyurethane films were investigated. When the mass ratio of (P + F)/PEG was 40/60, the tensile strength of prepared PU film PM-5 was 28.1 MPa that improved by 279.7% compared with tensile strength of PM-1 (7.4 MPa). Introducing a certain amount of phenol and formaldehyde to PEG structure could improve the char residue of PF-PEGs polyurethane composite films. The DMA results suggested that the PF-PEGs films with high contents of phenol and formaldehyde exhibited higher degree of cross-link density and *T*_g_ compared with PM-1. The XRD and TEM results indicated that crystallization and phase separation of PF-PEGs films, which would be conducive to explaining mechanical properties from microstructure. The synthesis mechanism of PF-PEGs polyurethane composite materials was revealed according to the analysis results in this study. Moreover, the research on composite films provides a theoretical base for the preparation of the rigid polyurethane foams based on phenolic resins.

## Data Availability

Reprints and permissions information is available at www.nature.com. Readers are welcome to comment on the online version of the paper. All data included in this study are available upon request by contact with the corresponding author Yongjun Zhao (zyjun2007@126.com) or Fuxiang Chu (chufxg@163.com).
