# Author Correction: Synthesis and characterization of polyethylene glycol-phenol-formaldehyde based polyurethane composite

**DOI:** 10.1038/s41598-020-61553-7

**Published:** 2020-03-10

**Authors:** Juan Liu, RiQing Chen, ChunPeng Wang, YongJun Zhao, FuXiang Chu

**Affiliations:** 1 College of Biological, Chemical Science and Engineering, Jiaxing University, Jiaxing, 314001 China; 2 Institute of Chemical Industry of Forestry Products, CAF, Nanjing, 210042 China; 3 Institute of Forest New Technology, CAF, Beijing, 100091 China; 4 Chinese Academy of Forestry, Beijing, 100091 China

Correction to: *Scientific Reports* 10.1038/s41598-019-56147-x, published online 20 December 2019

The original version of this Article omitted an additional affiliation for RiQing Chen, ChunPeng Wang and FuXiang Chu. The correct affiliations for these authors are given below:

RiQing Chen:

Institute of Chemical Industry of Forestry Products, CAF, Nanjing, 210042, China

Institute of Forest New Technology, CAF, Beijing 100091, China

ChunPeng Wang:

Institute of Chemical Industry of Forestry Products, CAF, Nanjing, 210042, China

Institute of Forest New Technology, CAF, Beijing 100091, China

FuXiang Chu:

Institute of Forest New Technology, CAF, Beijing 100091, China

Chinese Academy of Forestry, Beijing, 100091, China

This error has now been corrected in the PDF and HTML versions of the Article.

